# Synthesis, characterization and application of high adsorption performance of novel 1,4-polyketone

**DOI:** 10.1038/s41598-022-20686-7

**Published:** 2022-09-29

**Authors:** Marwa M. Sayed, Islam S. Abd El-Hamid, Haitham M. El-Bery, Mostafa Farrag, Alaa K. Abdelhakiem, Kamal I. Aly

**Affiliations:** 1 Chemistry Department, Faculty of Science, The New Valley University, El-Kharja, 72511 Egypt; 2 Polymer Research Laboratory, Chemistry Department, Faculty of Science, Assiut University, Asyut, 71516 Egypt; 3 Assiut Cement Company (Cemex Egypt), 18th Assiut- Elwadi Road, Asyut, Egypt; 4 Advanced Multifunctional Materials Laboratory, Chemistry Department, Faculty of Science, Assiut University, Asyut, 71515 Egypt; 5 Nanoclusters and Photocatalysis Laboratory, Chemistry Department, Faculty of Science, Assiut University, Asyut, 71515 Egypt; 6 Department of Pharmacy Practice and Clinical Sciences, University of Texas at El Paso School of Pharmacy, El Paso, TX USA

**Keywords:** Chemistry, Materials science

## Abstract

This study aims to develop an alternating polyketone containing cationic groups in one and four alternating positions for increased functionality. A novel polyarylidene ketone was synthesized using simple condensation polymerization of terephthaldehyde and 2,5-hexane dione (PAK) The physicochemical properties of the resulting polymer were evaluated using Fourier-transform infrared spectroscopy, thermogravimetric analysis, X-ray diffraction, UV-Visible absorbance, fluorescence, and SEM investigations. The findings show that the polymer is amorphous, has good thermal stability, and emits red light. It can also be used as a dye adsorbent in aqueous solutions, with high selectivity for the cationic dye methylene blue (MB). The adsorbent efficiency of PAK was measured as a function of pH, dosage, and initial dye concentration; the greatest dye removal of 96 % was obtained at pH 10, 50 mg dosage, and initial dye concentration of 20 ppm. Kinetics and isotherms were studied, showing that the pseudo-second-order model described kinetic data better than Freundlich and Langmuir and revealed a satisfactory chemisorption process. This study suggests that PAK can purify MB dyeing wastewater, remove Zn^2+^, Cu^2+^, Ni^2+^, Co^2+^, Cd^2+^, Fe^3+^ metal ions well, and is selective for Fe^3+^ and Cu^2+^; ion adsorption is chelating-based.

## Introduction

Water supplies are crucial for preserving a sufficient food supply and production ecosystem for each surviving organism. In addition to the expansion of population size and market demand, the worldwide need for clean water has increased rapidly. Effluent from manufacturing, agriculture, transportation, and houses threatens surface water. Many heavy metals, pesticides, hydrocarbons, and organochlorine compounds are discharged, leading to water pollution^1^. Many treatment procedures are used to remove harmful chemicals from water, including^2^ chemical, ion exchange, electrochemical treatment technology, physical and biological treatment, activated carbon, and adsorption methods^3–11^. Many polymers are used as adsorbents for waste from wastewater, and molecular imprint polymers (MIP) can be employed as ideal materials for effluent treatment^9,12^. Dye molecules are organic soluble compounds that dissolve easily in water, and their Removal is hard with conventional methods. This connection prevents light transmission through water, decreasing photosynthesis and dissolved oxygen, affecting the complete aquatic biota^13–15^.

Metals such as iron (Fe), nickel (Ni), manganese (Mn), zinc (Zn), cobalt (Co), chromium (Cr), magnesium (Mg), molybdenum (Mo), copper (Cu), and selenium (Se) are essential for a variety of biological and physiological processes. The deficient supply of these micro-nutrients causes insufficiency diseases or disorders^16^. Their existence in water is through mining and extracting various factors from their respective ores. Numerous procedures have been employed to get rid of serious metals from unclean water, such as membrane filtration, ion exchange, adsorption, precipitation^17^, reverse osmosis, solvent extraction, and electrochemical treating. Some of these techniques are costly in both operation and execution. The adsorption process is the most efficient and suitable due to its ease and minimal cost. Different adsorbents are utilized for this purpose, such as zeolite, activated carbon, clay minerals, organic polymers^18^, biochar, and several discarded materials, such as water treatment residuals and recycled sanding wastes and biomass.

Polyketones (Pks) are polymers that contain a random or alternate distribution of ketone groups along their principal chain. Eco-friendly aliphatic Pks are randomly distributed and formed from olefins and carbon monoxide^19,20^. Due to their limited use in life and the industry, scientists have not received them well^21^. Alternating Pks make up a very interesting class of polymers as their preparation is through copolymerization with the third monomer like styrene or long alkene^22,23^. They attract scientists owing to their ease of formation, high performance, multi-functionality, and applications like fibers^24,25^ and packing films^26^. The existence of substantially reactive 1,4-dicarbonyl groups in the chain make them a starting material to produce functional polymers that are not easy to prepare by conventional methods through various reaction pathways. The system can be easily converted to a wide variety of polymers^27^, including heterocyclic rings like pyrrole^19^, furan, thiophene, or groups such as bisphenol, alcohol^28^, ketal, and thiol. Aromatic Pks with aromatic rings along the chain are seldom prepared, although they have excellent chemical and physical properties^29^. Their limitation is restrained by incorporating ether links in their chains, which leads to diversity in their preparation. Thus, introducing an aliphatic segment between the aromatic rings gives some flexibility to the polymer chain and facilitates its application.

In continuation of our work in the synthesis and characterization of different kinds of polyketones (Pks)^30–33^, we report here in this article that a new polyarylidene-ketone (PAK) containing reactive 1,4-dicarbonyl groups from 2,5-hexane dione and terephthaldhyde that we have synthesized by simple condensation polymerization. We aim to take advantage of the reactive groups in the main chain in dye and heavy metal adsorption. The alternating 1,4-di-carbonyl functionality in the polymer backbone can serve as fantastic precursors for other functional polymers and substances with oxygen-containing functional groups that could result in certain unique adsorption features. As the substance's hydrophilicity can be increased by the presence of oxygen-containing groups, the contact area between it and the dye molecules in water can be increased. Additionally, the oxygen-rich units may make the functionalized polymer more electronegative, providing opportunities for extra electrostatic interactions with cationic dye molecules and metals. For instance, methylene blue, a positively charged cationic dye, and the lone pair of electrons on the O atom interact electrostatically. Even though recent research suggests that adding oxygen-containing groups may considerably impact adsorption efficacy^34^. The prepared polymer shows high thermal stability, fast adsorption affinity towards methylene blue (MB) dye, and substantially eliminates heavy metals from water samples.

## Experimental

### Reagents and solvents

All chemicals used in this study are analytical grade and used without further purification, including potassium carbonate (K_2_CO_3_), potassium hydroxide (KOH), sulfuric acid (H_2_SO_4_), and nitric acid (HNO_3_).

Other chemicals include 2,5-Hexandione and terephthaldehyde from (Alfa Aeser), sodium hydroxide, anhydrous ferric (III) chloride, zinc acetate, copper (II) acetate hydrate, cobalt (II) chloride hexahydrate, and nickel (II) sulfate hexahydrate from (Fisher Scientific), methylene blue dye (Merck-Schuchardt), organic solvents acetone, ethanol, dimethyl sulfoxide (DMSO), dimethylformamide DMF are retrieved from Sigma-Aldrich and employed without extra distillation.

### Measurements

Shimadzu 2110 PC Scanning Spectrophotometer is used for the measurement of infrared Spectra. Using flame and graphite atomization techniques, the atomic absorption is measured on a Buck model 210 VGP Inc. East Norwalk, CT (USA). Bruker D8 Advance for X-ray diffractograms of polymers, copper closed tube with X-ray unit, and kα radiation at a wavelength of 1.5406 Å is produced from an x-ray source. TA 2000 thermal analyzer and Shimadzu DTG-60 were utilized for TGA and DTG measurements at a heating rate of 10°C/min under N_2_. Scanning electron microscope (SEM) Joel- JSM-5400 LV was employed to examine polymers morphology. The polymer sample is coated with a gold-palladium alloy for SEM examination on a copper holder. Nova 3200 surface area instrument for BET data under nitrogen atmosphere is also utilized. A Perkins-Elmer UV-VIS spectrophotometer is utilized for UV-Vis spectra measurements, and the sample is placed in a 1 mm quartz cell.

### Synthesis of poly arylidene ketone (PAK)

**Figure 1:**
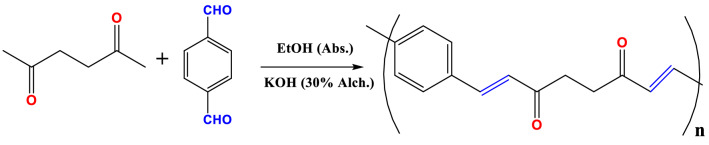
Synthesis of PAK.

PAK is synthesized in a high yield by polymerizing terephthaldhyde with 2,5-hexanedione in a basic ethanolic solution using an aldol condensation reaction^35–37^ (Fig. 1). In a two-neck flask, terephthaldehyde (0.0015 mmoles, 0.2 gm) is completely dissolved in about 50 ml of absolute ethanol, then 2,5 hexanedione (0.0015 mmoles, 0.17 gm) in 10 ml of ethanol 95% is added into the mixture and nitrogen gas inlet-outlet. After about 15 minutes, a few drops of 30% KOH alcohol are supplied as a basic media, and the mixture was agitated at a steady temperature of 70 °C for another 15 minutes. The solution's viscosity rapidly increases, and the polymer precipitated early in the reaction. Filtration is used to recover the generated polymer, which is then cleaned with water and ethanol and dried in an oven at 80 °C.

### Preparation of methylene blue dye solution

The standard solution of methylene blue is made in pure water at ambient temperature with a concentration of approximately 130 ppm at pH 9, and a solution of (5 × 10^− 2^ M) NaOH and (5 × 10^− 2^ M) HCl is prepared to control the pH of the solution. The calibration curve is created by organizing a series of diluted solutions from the stock solution.

### Preparation of single dye solution containing adsorbent

Different weights of dried polymer adsorbent (5–50 mg) from PAK samples are added to 200 ml of dye solution with varying concentrations and pH. Then, approximately 5 ml of aliquot parts are transferred using a syringe, and UV spectroscopy is used to determine the concentration at various intervals.

### Preparation of heavy metal solutions

The stock solution of heavy metals utilized, which included Zn^2+^, Cu^2+^, Ni^2+^, Co^2+^, Cd ^2+^ and Fe^2+^, has a concentration of 20 ppm in pure water at room temperature.

## Results and discussion

**Figure 2:**
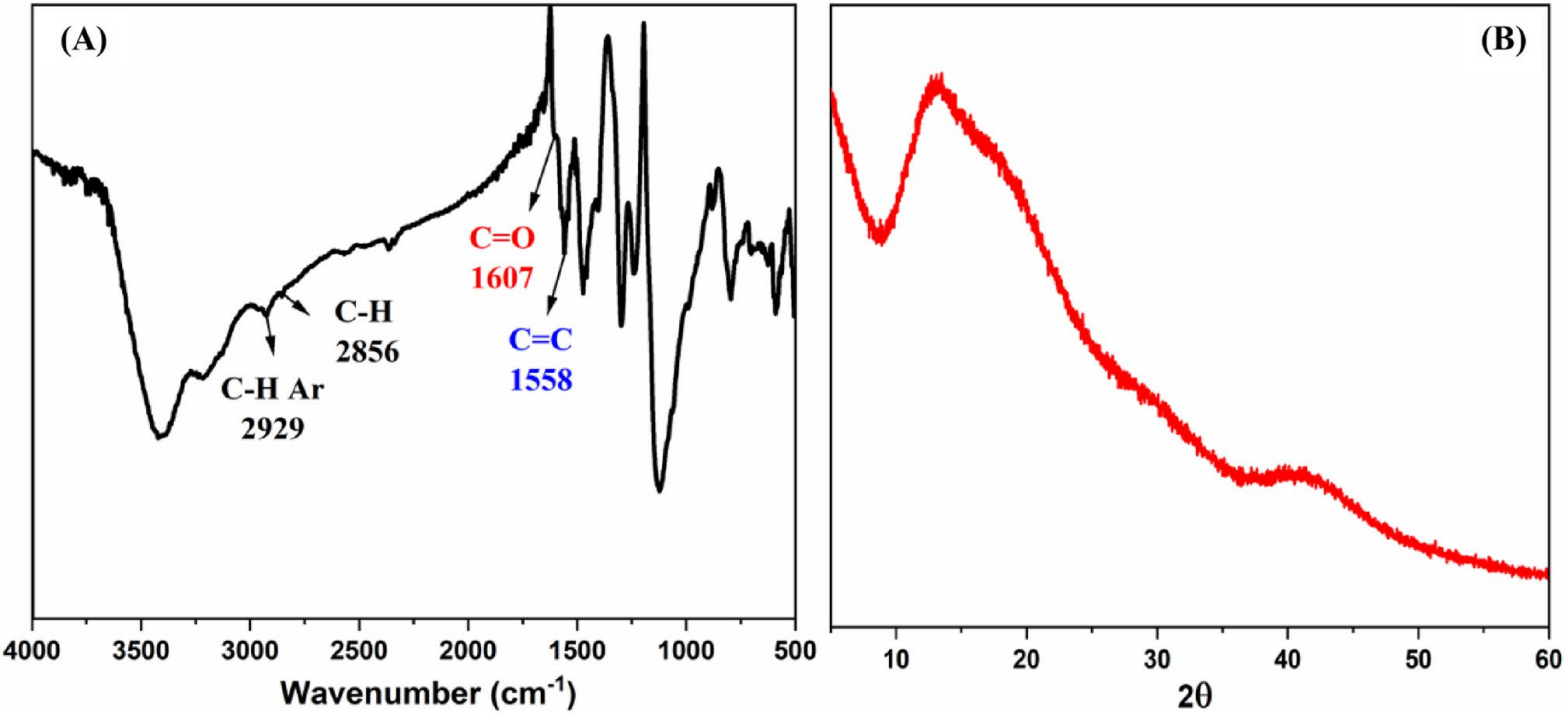
(**A**) FT-IR spectrum, (**B**) X-ray graph of PAK.

The structural property of unsaturated aliphatic-aromatic polyketone PAK provides an opportunity to manufacture polymer utilizing simple monomers rather than two co-monomers using an aldol condensation technique, which results in a higher yield. Several distinct spectroscopy methods are used to characterize the structure of the polymer. The FT-IR spectrum of PAK (Fig. 2A) displays the characteristic bands for the C=O stretch vibration at 1607 cm^−1^ with reduced intensity, conjugated C=C bond at 1558 cm^−1^, C-H aliphatic at 2856 cm^−1^, and C-H Aromatic at 2929 cm^−1^. Due to a large amount of the CO groups, conjugation, and alternating distribution, the wavenumber of the carbonyl group has changed to a lower value, and the peak intensity has dropped. More stacking of molecules in semicrystalline structure and greater intermolecular force resulted from this uniformity in allocation^38^. These findings point to polymer formation, structure elucidation, also physical and chemical behavior.

The crystallization behavior of polyketone was studied using the X-RD method. As demonstrated in Fig. 2B, PAK has a semi-wide peak at 2θ = 13.14°, indicating some degree of crystallinity. The alternating arylidene moieties between the aliphatic chain and ketonic groups provide the polymer structure with some regularity and result in higher crystallinity (56.7 %) than amorphous^39,40^. This unique structure affects the chemical and physical properties of the resultant polymer, making it more resistant to solvent penetration and heat breakdown. Mechanical, optical, chemical and thermal properties are all affected by a material's degree of crystallinity. The PAK chain comprises aromatic rings and ketone groups alternately linked together. The crystal's strength is increased due to the aromatic rings' tendency to stack neatly. Several driving factors (electrostatic attraction, hydrogen bond, ion exchange, and "stacked aromatic rings) can be used to synthesize adsorbers to boost the adsorption rate and reduce the time needed to reach equilibrium adsorption; these functional groups provide adsorption sites for dye molecules.

The resulting PAK's solubility was examined, showing that the polymer is not soluble in polar protic solvents but partially soluble in a polar aprotic solvent like DMSO and DMF. This can be attributed to its structure and the high polarity content of the carbonyl group.

**Figure 3:**
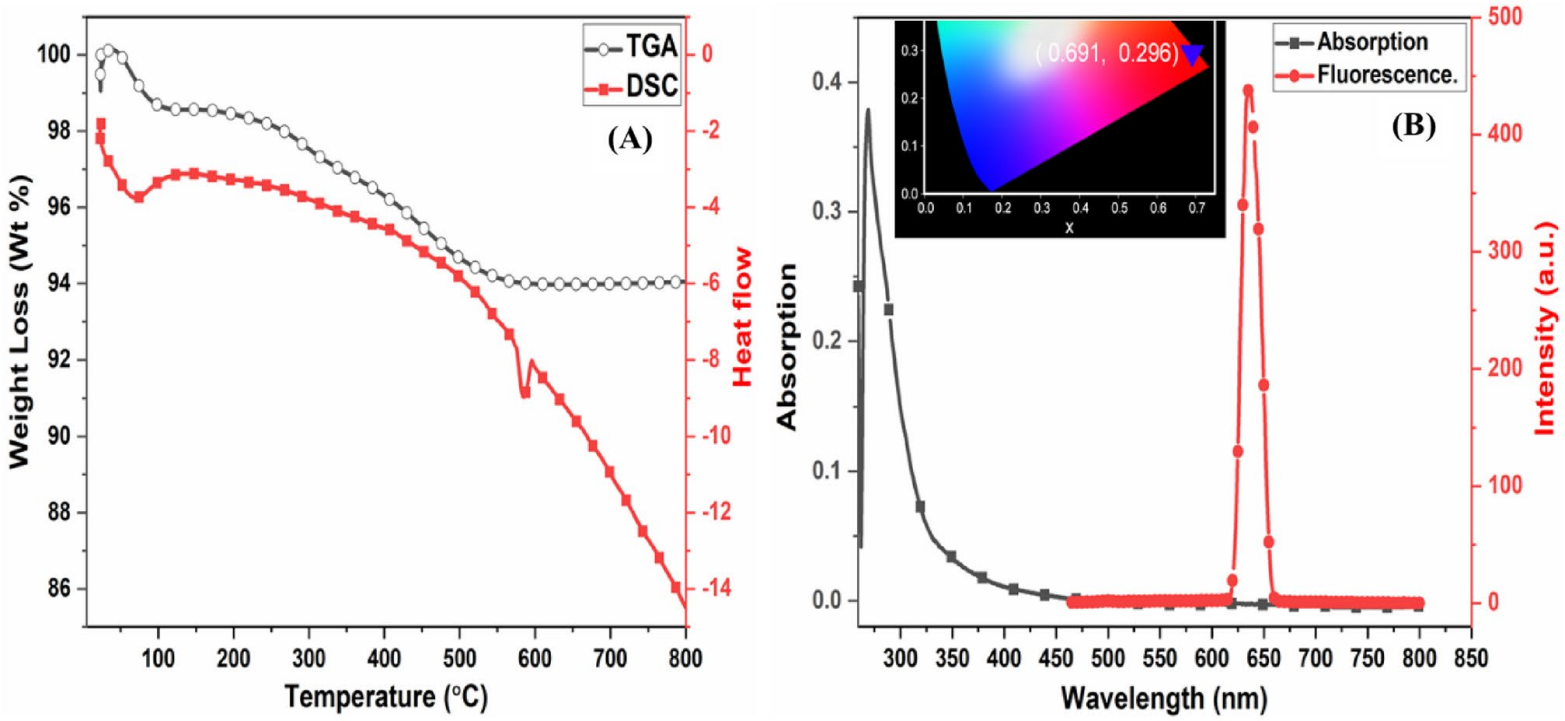
(**A**) TGA-DSC spectra, (**B**) Uv- visible spectrum, emission spectrum at λ_exc._ 320 nm, and the inset photo shows the CIE diagram of PAK.

Thermogravimetric analysis TGA and differential scanning calorimetry DSC curves evaluated PAK's thermal stability (Fig. 3A). The DSC results show that the polymer has a high melting temperature of *T*_*m*_ at 580 °C and a glass transition temperature of *T*_*g*_ at 74 °C, which is beneficial because thermal stability is mainly dependent on crystalline structure increases with crystallinity. The vaporization of trapped solvent and moisture in the polymer causes a weight loss of around 1.4% at temperatures below 100 °C. In contrast, the gradual weight loss in the second stage of decomposition is fast, about 6 % from 110 to 560 °C due to the decays of PAK chains. Furthermore, at 700 °C, the char yield is 93.9%. These findings support the existence of PAK polymorphisms with excellent thermal stability and crystalline structure^41^.

The photophysical features of PAK's UV-visible absorption and emission spectra are discussed in Fig. 3B. An absorption band at λ_max_ 269 nm may be seen in the absorption spectrum of PAK solution (1 mg in 10 ml DMF). This peak could be attributed to the π-π^*^ transition of arylidene conjugation with the carbonyl group of the polymer backbone, as aliphatic polyketone solutions are colorless and do not absorb UV light due to the lack of conjugation in their chains and the presence of the ketonic group^42^. While the emission spectrum of solid PAK with λ_exc._ 280 nm exhibits a peak at λ_max_ 511 nm, a higher intensity in the red region; the emission color is determined by the Commission International de l'Elcairage CIE chromaticity diagram with (x,y) coordination (0.691, 0.296), indicating red color emission. This emission can be linked to PAK's crystal structure and improved interchain stacking in the solid-state, resulting in this emission^43^.

**Figure 4:**
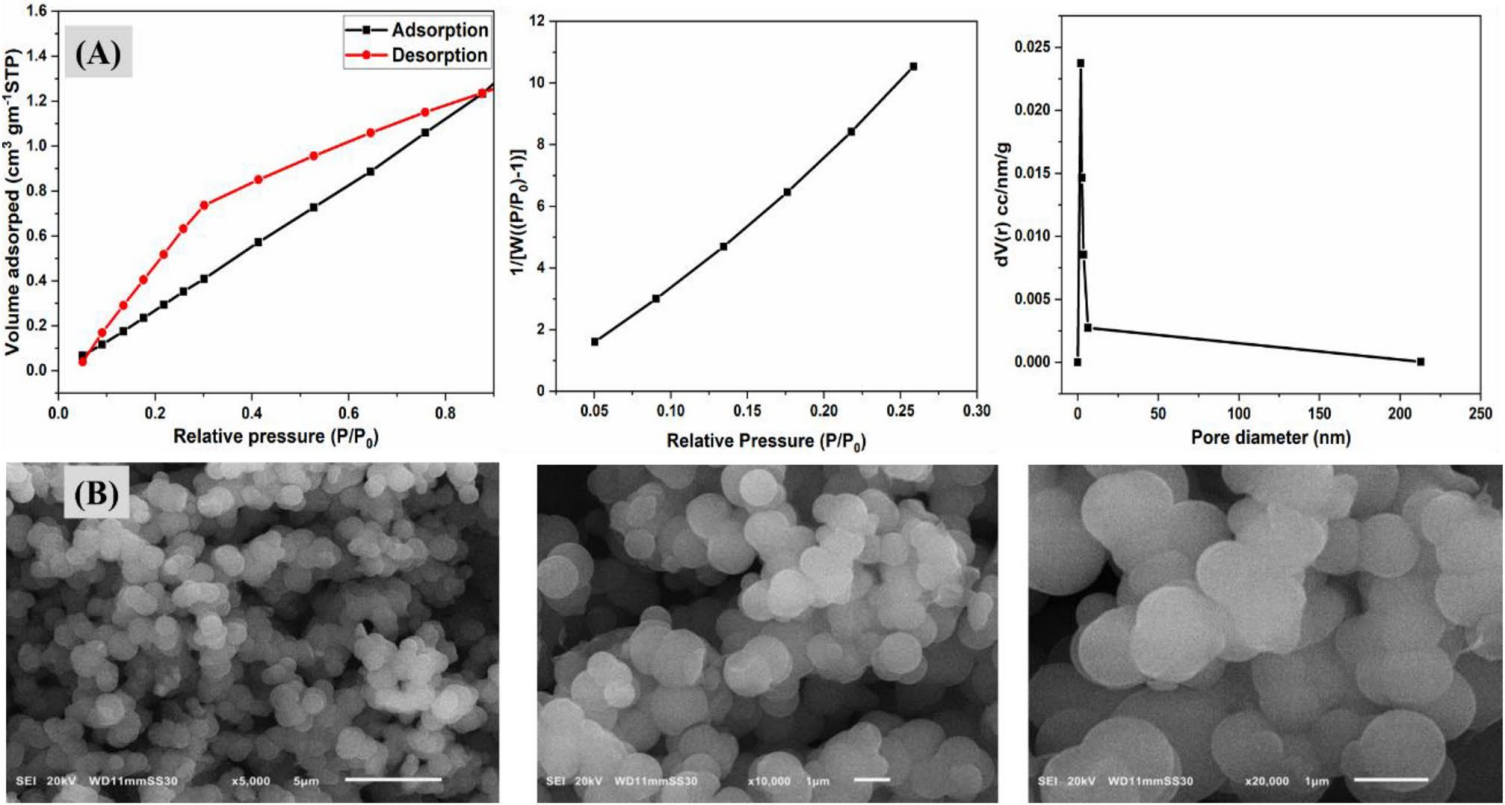
(**A**) BET nitrogen adsorption isotherm plot BET surface area and pore size distribution, (**B**) SEM-EDX images at different magnifications of PAK.

Gas sorption analysis with N_2_ was used to study the porous structure of this PAK. Figure 4A depicts the resultant polymer's N_2_ adsorption and desorption data. The BET surface area appears to be 76.73 m^2^/g. The pore volume of the polymer is 0.0687 cc/g. The pore diameter of the polymer is 1.7911 nm, indicating that it is extremely close to being microporous^44^. Polymer density (or concentration) affects accessible surface area but not chain length.

Furthermore, the available concentration of smaller molecules in thick, brittle bendable polymers can be as high as 10^-3^. When chains have low flexibility and cannot compress and versatile chain integration, the total area available may be directly proportional to the twisted chain portions that hinder the chains from collecting and producing permeable pieces. Due to the packing, adjacent chains would come into contact, preventing the probe from touching the contact surface and lowering the surface area that could be accessed. Because the arylidene moieties are present, the surface area of this PAK is reduced. In these conditions, the arylidene effectively fills a convex region on the molecule's surface^45,46^.

Enlarging the size of an object and allowing individuals to observe smaller sections within the sample is a useful approach to inspecting the surface of the polymer. Figure 4B shows the morphology of PAK as seen using a scanning electron microscope (SEM) at various magnifications. This information has been utilized to track bulk polymers' crystal shape, structure, and some of their mechanical and optical properties. The spherical form with coalescence is founded in the SEM analysis of PAK. The crystallinity of PAK, the ordering between chains, the interchange distance, and the increased porosity are all due to the compact packing of globules, which has several uses. The presence of the flexible aliphatic chain allows for accumulation and packing between chains^47,48^.

Pyrolysis GC/mass was used to determine the composition of a PAK sample, which was pyrolyzed at 600 °C to ascertain its design. The pyrolysis temperature and the pyrograms (total ion chromatograms) are displayed in Fig. S1. The breakdown products indicate the presence of a monomeric unit of the polymer at m/z 343.2. At 650 °C, the breakdown product suggests a dimer unit with m/z 646.5. These findings point to one of the most widely used domains in gas chromatography: constructing a polymer chain and examining its greater thermal stability in the face of heat.

### Analysis of the factors affecting adsorption

**Figure 5:**
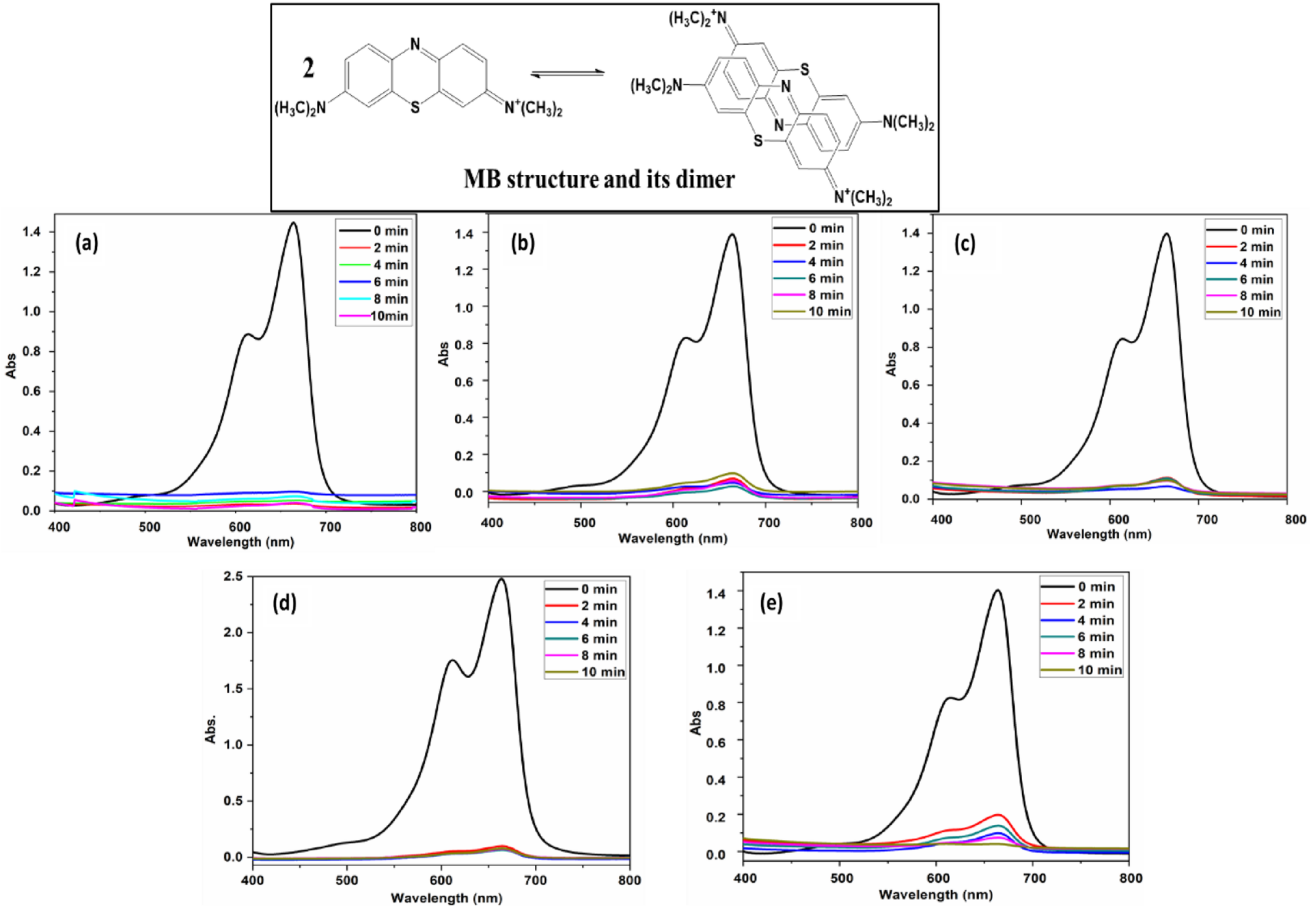
UV–Vis spectra of 20 ppm (MB) solution adsorption (**a**, **b**, **c**, **d**, **e**) were recorded for (10, 20, 30, 40, 50 mg) different weights of PAK at pH 10.

Methylene blue (MB) is a triphenylmethane-related acid cationic dye Fig. 5, and it is used to color cotton, wood, and silk. Possibly cause lasting eye burns on human and animal eyes. The UV-visible absorption spectra of MB in water exhibit bands at 664 nm, 612 nm, and 556 nm, which correspond to monomers (MB^+^), dimers [(MB^+^)_2_], and higher aggregates [(MB^+^)_n_]. Hydrophobic contacts, hydrogen bonding, Van der Waals forces, London dispersion forces, and other short-range forces hold dimers, trimers, and higher aggregates together. MB shows absorption at 612 and 666 nm according to monomer/dimer equilibrium and is concentration-dependent; increasing MB concentration lowered the 666/612 nm absorbance ratio^49^.

Adsorption kinetics were investigated at various intervals to measure the amount of MB dye adsorbed. We used 200 ml of dye solution with concentrations extending from 15 to 100 ppm, continuous stirring at 700 rpm; 2 ml from the stirred sample was collected every 2 minutes between each patch within a 10-minute time interval and then filtered with filter paper before analyzing with a quartz cuvette using UV/visible spectrophotometry at λ_max_= 660 nm. Several parameters (polymer dosage, pH, and beginning dye concentration) are tested to discover the most effective circumstances for increasing the quantity of MB dye adsorbed.

The equation below calculates the adsorption efficiency percent (AE %) of MB on polymer^50^:

1 $${\text{AE}}\% \; = \;\frac{{\left( {C_{0} - C_{t} } \right)}}{{C_{0} }}*100$$

*C*_*0*_ and *C*_*t*_ are the beginning MB dye and dye concentration at period *t* (mg/l). The Beer-Lambert law determines the remaining concentration of MB dye. The *q*_*e*_ and *q*_*t*_ of the dye adsorbed are given by the Eqs. (2, 3):

2 $${\text{q}}_{{\text{e}}} = \frac{{\left( {C_{0} - C_{e} } \right){\text{V}}}}{m}$$

3 $${\text{q}}_{{\text{t}}} = \frac{{\left( {C_{0} - C_{t} } \right){\text{V}}}}{m}$$

where s*q*_*e*_ is the adsorbed quantity at equilibrium for MB dye, *q*_*t*_ is during time *t*, the amount of MB dye adsorbed at equilibrium, and both *q*_*e*_ and *q*_*t*_ have the unit (mg g^-1^).

#### Effect of adsorbent mass (quantity)

This study aimed to see how the adsorbent dose affected the results at a constant temperature of 298 K and a fixed concentration of MB of 20 ppm, the polymer dose in 200 ml was increased from 10 to 50 mg. Adsorption behavior was studied by obtaining a sample every 2 minutes for 10 minutes. The data revealed that when utilizing 40 and 50 mg as polymer doses, the polymer's adsorption effectiveness (AE %) was approximately 96%. When using 5 mg of polymer, 66% AE was also obtained (Fig. 5). The increased number of accessible active pore sites for adsorption and higher specific surface area are the reasons for boosting the AE of MB from 66 to 96% by increasing the polymer dosage^51^.

The dose's impact may be understood by evaluating the adsorbent's particle size. 0.2 mg/ml of PAK is dissolved in distilled water, and the Malvern Zetasizer, Nano ZS series, is used to examine the results. After repeating the measurement, the z-average was determined to be the particle average size for the initial test run, according to Fig. 8a. The study demonstrated that the size distribution by intensity includes two peaks measured at 2227 nm peak 1 and 280 nm peak 2. The non-uniformity of the particle size results in a polydispersity index (PDI) value of 0.381 and total z-average measurement of 4463 nm. The size of the particles increases when the sample is diluted, and the size distribution contains one peak at 5560 nm with a z-average of 9605 nm and PDI 0.850. This can be explained by the fact that, for a given amount of adsorbent, a reduction in particle size can increase surface area availability, causing nano aggregation, which increases the number of active sites for dye adsorption^52^.

#### Effect of initial dye concentration

**Figure 6:**
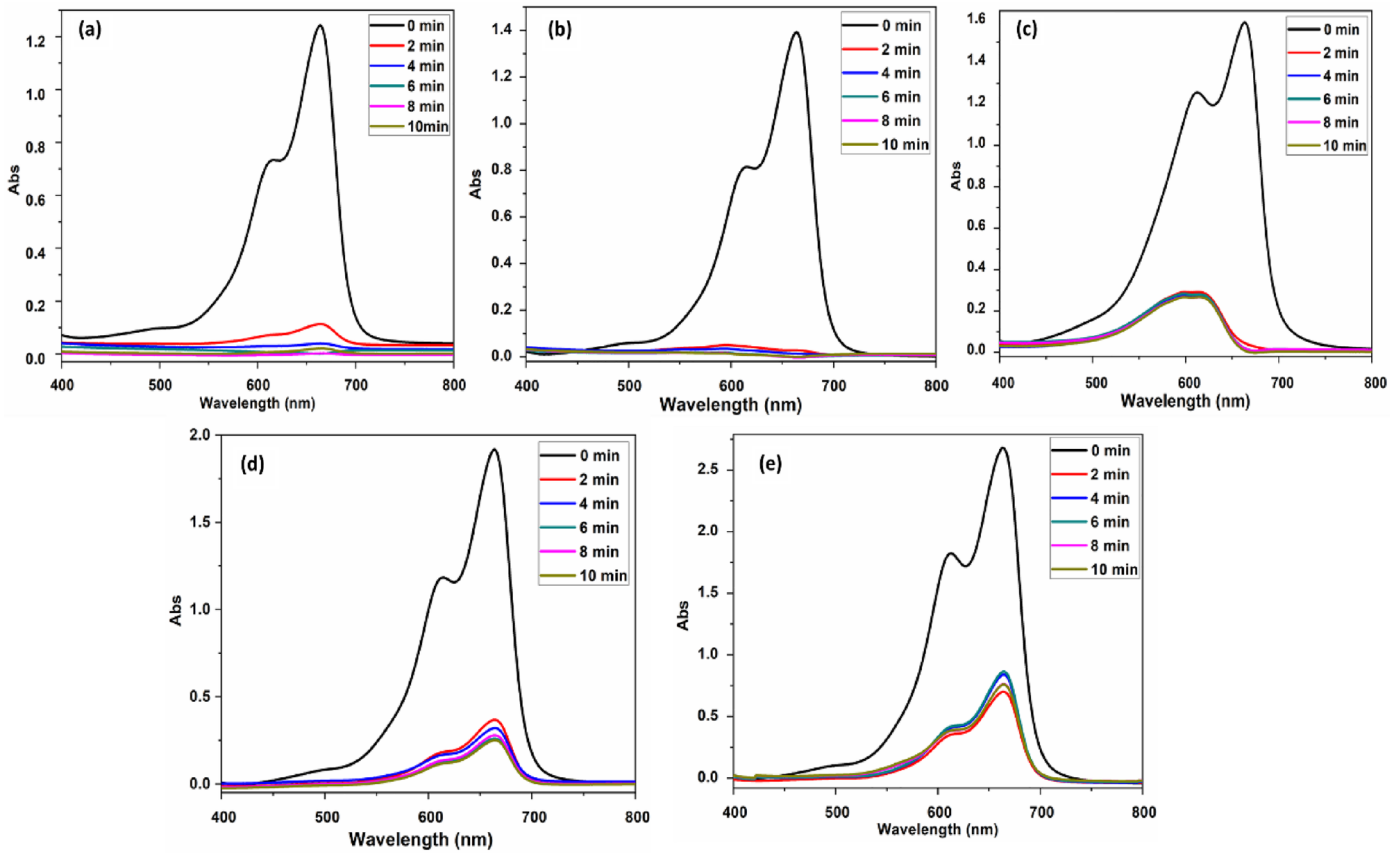
UV–Vis spectra of (MB) solution adsorption at different concentrations (**a**, **b**, **c**, **d**, **e**) were recorded for (15, 30, 40, 65, 100 ppm) of 40 mg PAK at pH 10.

All other variables and parameters, such as pH, temperature, and polymer dose, were constant, except for the MB beginning dye concentration, modified to emphasize the relationship between initial dye concentration and AE % (Fig. 6). When the first dye concentration was elevated from 15 to 30 ppm at pH 10, the AE % of the 40 mg polymer was altered considerably. When the first dye concentration was raised from 40 to 100 ppm, the AE % decreased from 92 to 83%. Because we employed a certain dose of polymer in our research, the number of accessible sites on the polymer's surface is fixed. Consequently, the number of places is constant. As a result, the ratio of active and accessible sites on the polymer surface to the MB dye molecule is always larger than in lower concentrations. However, the available active site on the polymer's surface was occupied at higher dye concentrations, reducing the AE %. As a result, the active site to MB dye molecule ratio is below one^53^.

#### Effect of pH

**Figure 7:**
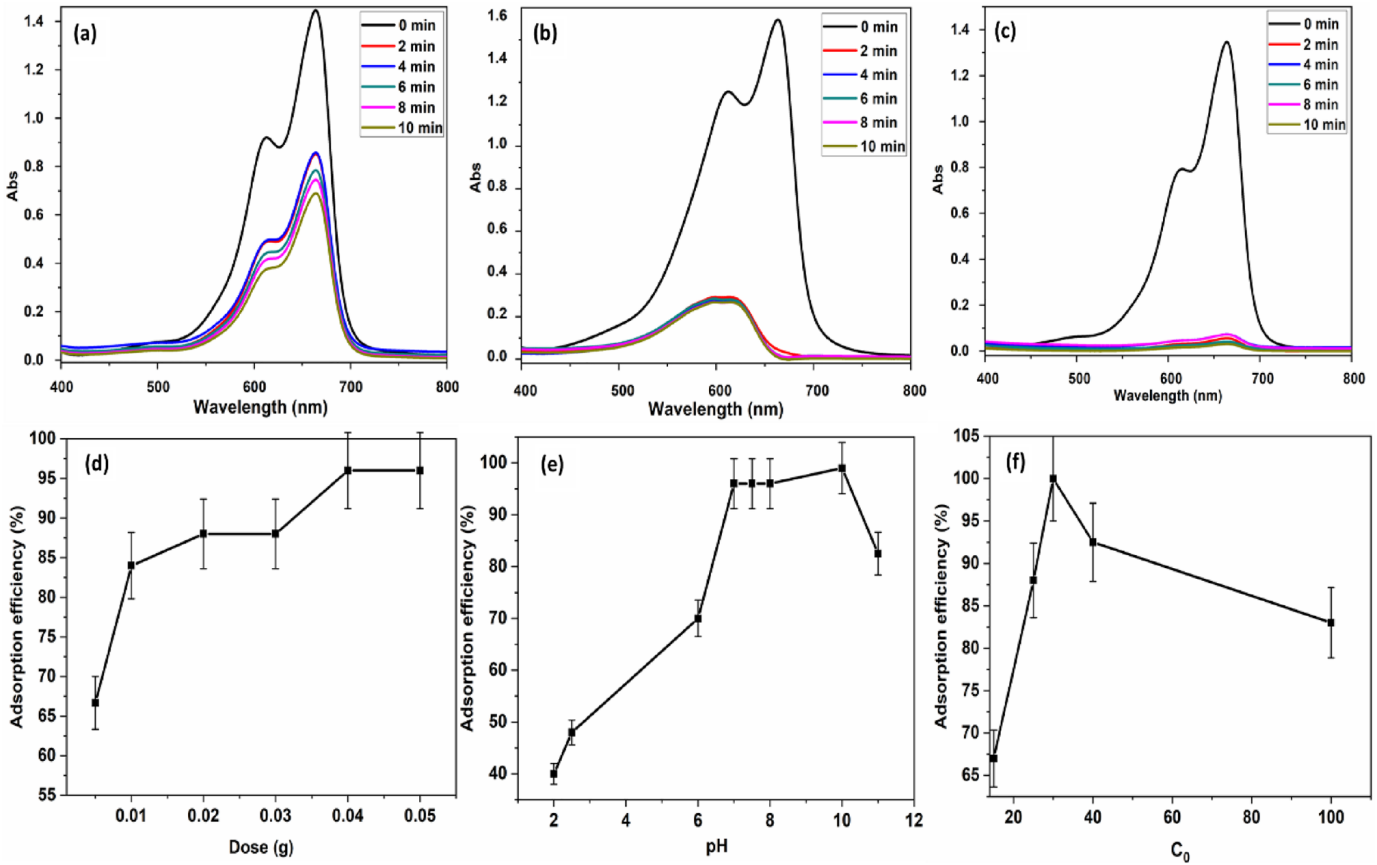
(**a**, **b**, **c**) Show UV–Vis spectra of 20 ppm MB solution before and after mixing with 40 mg PAK in conjunction with pH change (**a**) pH 2.5, (**b**) pH 7.5, (**c**) pH 10**.** (**d**, **e**, **f**) Show the relation between adsorption efficiency % and (**d**) dose of polymer, (**e**) pH change, and (**f**) initial dye concentration of MB.

**Figure 8:**
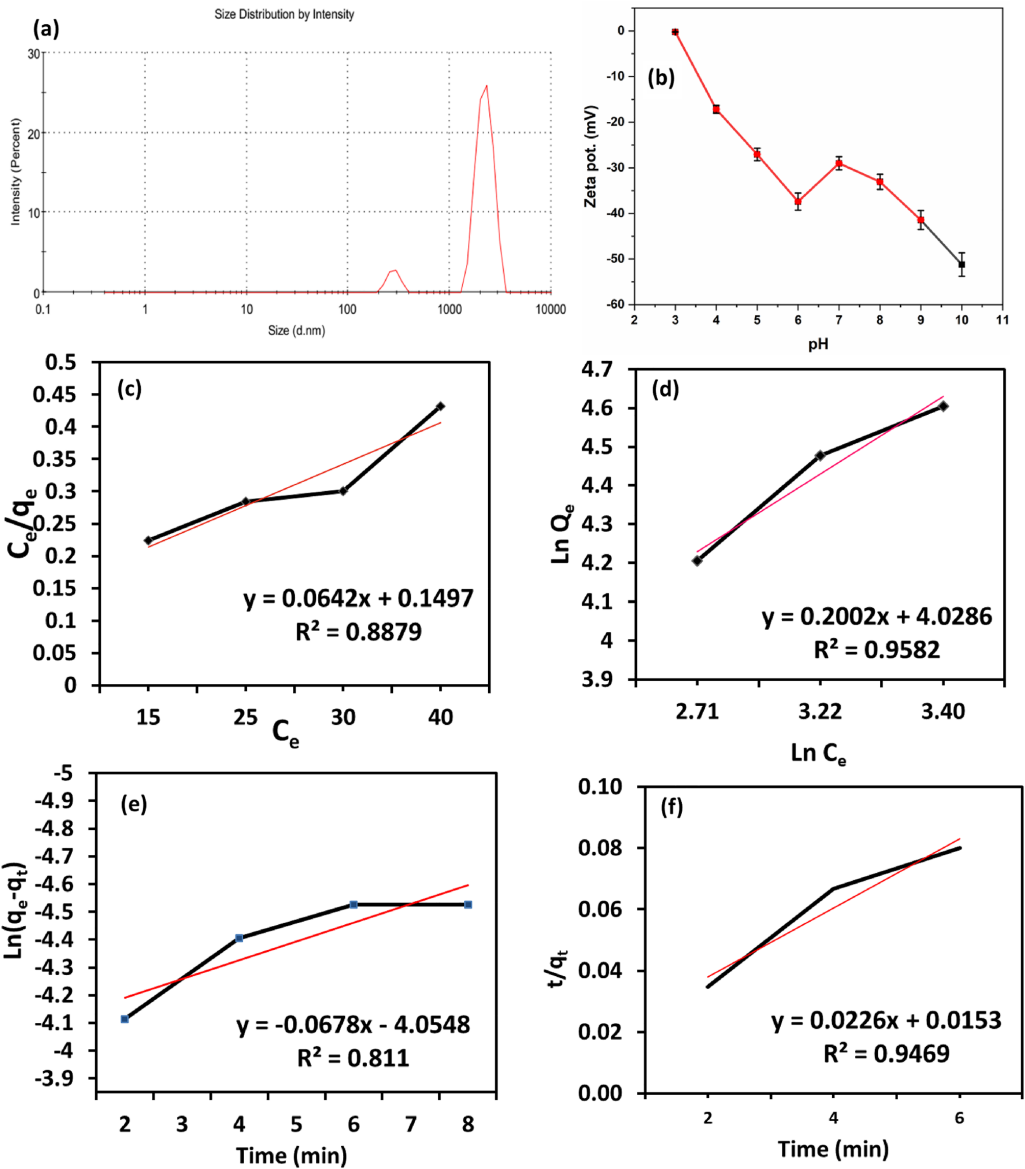
(**a**) Particle size analysis of PAK, (**b**) Zeta potentials of PAK, (**c**) Langmuir, (**d**) Freundlich, (**e**) Pseudo First Order (PPO), and (**f**) Pseudo-second Order (PSO) isotherm models for MB adsorption onto PAK.

The pH of the MB solution is a significant component that affects adsorption capacity directly. The adsorption efficiency of the adsorbent is evaluated using the pH of the MB solution. In this study, we changed the pH of the solution while keeping a constant temperature of 298 K and an amount of adsorbent that is constant (40 mg) in 200 ml of dye solution with a concentration of 20 ppm (Fig. 7a–c)^54^. MB is a positively charged molecule and includes aromatic rings, the electrostatic attraction between MB molecules and PAK is constrained by factors such as the pH of the adsorbing solution^55,56^. Therefore, only in a specific pH range can optimal adsorption performance be attained, and it is important to examine adsorption performance that is unaffected by the surrounding atmosphere. The presence of aromatic rings in the PAK and MB dye molecular structures and stacking interactions between the two molecules must be considered^57,58^. Because of the combined favorable effect of stacks and electrostatic attraction, removal efficiency generally declined as pH rose from 3 to 7; nevertheless, electrostatic attraction should be considered. The reason is that between pH 7 and pH 10, PAK demonstrated weaker zeta potentials and isoelectric point at pH 3 Fig. 8b. More crucially, the adsorption behavior in alkaline solution was practically independent of pH, and this is all due to the crucial role performed by stacking interactions. Adsorption effectiveness is highest and most stable between 8 and 10 pH^59^. The previous variables are summarized in Fig. 7d–f to indicate their effects on adsorption efficiency.

### Adsorption kinetics

#### Langmuir and Freundlich models

We employ the Langmuir and Freundlich models to validate our findings and better comprehend the research results. According to the Langmuir isotherm kinetic model, adsorption happens on a uniform surface of a monolayer material, and all adsorption points are equal and equivalent^60^. The Langmuir model has the following formula.

4 $$\frac{{C_{e} }}{{q_{e} }} = \frac{{C_{e} }}{{q_{m} }} + \frac{1}{{\left( {Kl*q_{m} } \right)}}$$

where *Ce* denotes the MB equilibrium state (mg l ^−1^), the equilibrium quantity adsorbed *q*_*e*_ (mg g ^−1^), the high adsorption capacity *q*_*m*_ (mg g ^−1^), and KL is the adsorption equilibrium constant (l mg ^−1^).

We use the second assumption in this analysis, the Freundlich isotherm model, which expects adsorption on diverse multilayer adsorbent material surfaces. The adsorbent material and the adsorbate have significant interactions, unlike the Langmuir isotherm type. The Freundlich isotherm type is expressed as:

5 $$\ln \;q_{e} = \ln \;Kf + \frac{{\ln C_{e} }}{n}$$

*C*_*e*_ and *q*_*e*_ are identical to the *C*_*e*_ and *q*_*e*_ in the Langmuir model. *Kf* is the Freundlich characteristic factor, closely linked with adsorption ability, and *n* is the adsorption strength.

The Langmuir and Freundlich parameters were obtained by graphing *C*_*e*_*/q*_*e*_ on the x-axis versus *C*_*e*_ on the y-axis and charting ln *q*_*e*_ on the x-axis versus ln *C*_*e*_ on the y-axis for both models (Fig. 8c,d). The MB dye adsorption process on the polymer surface indicated in the data was fit using both models (Table 1).

**Table 1:** Isotherm parameters for MB adsorption onto PAK at 298 K.

Langmuir model		Freundlich model	
C_e_/q_e_= C_e_/q_m_ + 1/kLq_m_		ln(q_e_)= lnKF+(1/n) ln(C_e_)	
q_m_ (mg g^−1^)	15.652	n	4.995005
KL (mg g^−1^)	0.426	KF (mg g^−1^)	56.182201
R^2^	0.88	R^2^	0.9582
y = 0.0642x + 0.1497		y = 0.2002x + 4.0286	

The results suggest that the Freundlich pattern is the best approximation for the polymer's MB dye adsorption process, with correlation coefficients of 0.88 and 0.9582, respectively. The Freundlich constant *Kf* was likewise found to have a high value, corresponding to a sorption capacity of 56.2.

Furthermore, the adsorption intensity was indicated by *n*, which is applied to evaluate the feasibility of the adsorption action. If *n* is 2-10, effective adsorption is displayed; if *n* is 1–2, moderate to difficult adsorption is indicated; if *n* is <1, poor adsorption is indicated^61^. The result of the *n* polymer is 4.99, showing that MB is easily adsorbed onto the produced polymer, as previously reported.

### Pseudo first order (PPO) and pseudo-second order (PSO)

The amount of MB adsorbed on the polymer (*q*_*t*_) for 10 minutes is investigated. After 2 minutes, the MB dye was rapidly eliminated, with roughly 90% of the dye gone. This result was due to a free and active adsorption site in the initial reaction stage that MB dye molecules could occupy. The removal effectiveness falls as the available active sites on the polymer surface diminish with time until all reactive points on the polymer surface are blocked and saturated with MB dye.

We used pseudo-first- and pseudo-second-order reaction kinetic techniques and both models to test our findings and further understand the peculiarities of the adsorption mechanism. The liner formula is expressed in Eqs. (6) and (7) for pseudo-first-order and pseudo-second-order kinetic systems, respectively (Fig. 8e,f).

6 $$\ln \;\left( {{\text{q}}_{{\text{e}}} - {\text{q}}_{{\text{t}}} } \right) = \ln q_{e} + Kl*t$$

7 $$\frac{t}{{q_{t} }} = \frac{t}{{q_{t} }} + \frac{1}{{K2\;\;q_{e}^{2} }}$$

where *q*_*e*_ is the adsorbed amount at balance for MB dye, *q*_*t*_ is the adsorbed quantity of MB dye at equilibrium at time *t* (mg g^-1^), and *Kl* is the constant of pseudo-first-order rate (min^-1^), and *k*_*2*_ is the constant of pseudo-second-order rate (g mg^-1^ min^-1^).

Straight-line charts illustrate both models' data, kinetic parameters, and correlation coefficient (R^2^) (Table 2). The pseudo-second-order model had higher linearity of correlation coefficient (0.9469) than the pseudo-first-order model versus (0.811), suggesting that the pseudo-second-order model provides the most accurate estimations of reaction kinetics in our manufactured polymer. The maximal adsorption capabilities of PKA and other adsorbents for MB are listed in Table 3. As shown, the adsorption capacity attained in this investigation was significantly higher than previously reported in the literature. This finding indicates that the PKA polymer has a high potential for MB removal^62–71^.

**Table 2:** Kinetic parameters for the adsorption of MB dye on PAK.

Pseudo-first order model		Pseudo-second order model	
ln(q_e_ − q_t_) = lnq_e_ – kl*t		t/q_t_=(1/k2q_e_^2)+t/q_e_	
q_e.c_ (mg g^− 1^)	57.67	q_e.c_ (mg g^−1^)	44.25
k1 (min^− 1^)	0.0678	k2 (mg g^−1^ min^−1^)	0.033
R^2^	0.811	R^2^	0.9469
y = − 0.0678x − 4.0548		y = 0.0226x + 0.0153

**Table 3:** The maximum adsorption capacities of PKA and other adsorbents for MB.

Adsorbents	Pollutant	Adsorption capacity (mg/g)	Referances
PPy/SD	MB	34.36	56
G an GO	MB, MV, RB, OG, Pb(II), Cu(II), Cd(II), Zn(II),	17.3	57
PANINTs/Silica	MB	25	58
Fe3O4@PAmA-BAmPD-TCATS	MB	31.64	59
SW-ZnO-PANI	MB	20.6	60
PProDOT/MnO2	MB	13.94	61
potato (Solanum tuberosum) plant	MB and MG (malachite green)	52.6	62
AC	MB	47.62	63
SNCM	MB	20.0	64
Fe2O3-ZrO2/Black cumin	arsenic and	38.1	65
PAK	MB, Cu(II), Cd(II), Zn(II),	57.67	This study

### Heavy metal removal studies

Many types of heavy metals have significant toxicity and are non-biodegradable, which leads to a highly bad impact on the health of humans, the environment, and animals. Therefore, removing heavy metals from sewer water is a big problem and a station for everyone's worldwide attention. Many researchers and technologies work to develop the removal process like membrane filtration, reverse osmosis, electrochemical treatment, extraction, and irradiation.

In this article, we investigate a polymer's capacity to remove heavy metals based on the presence of polyketones as functional groups that can generate more chemical than physical adsorption interactions. At pH 6, we created our study on the reduction of metal (Zn^2+^, Cu^2+^, Ni^2+^, Co^2+^, Cd ^2+^ and Fe^2+^) from wastewater as one of the effective methods used recently by the polymer in wastewater treatment.

**Figure 9:**
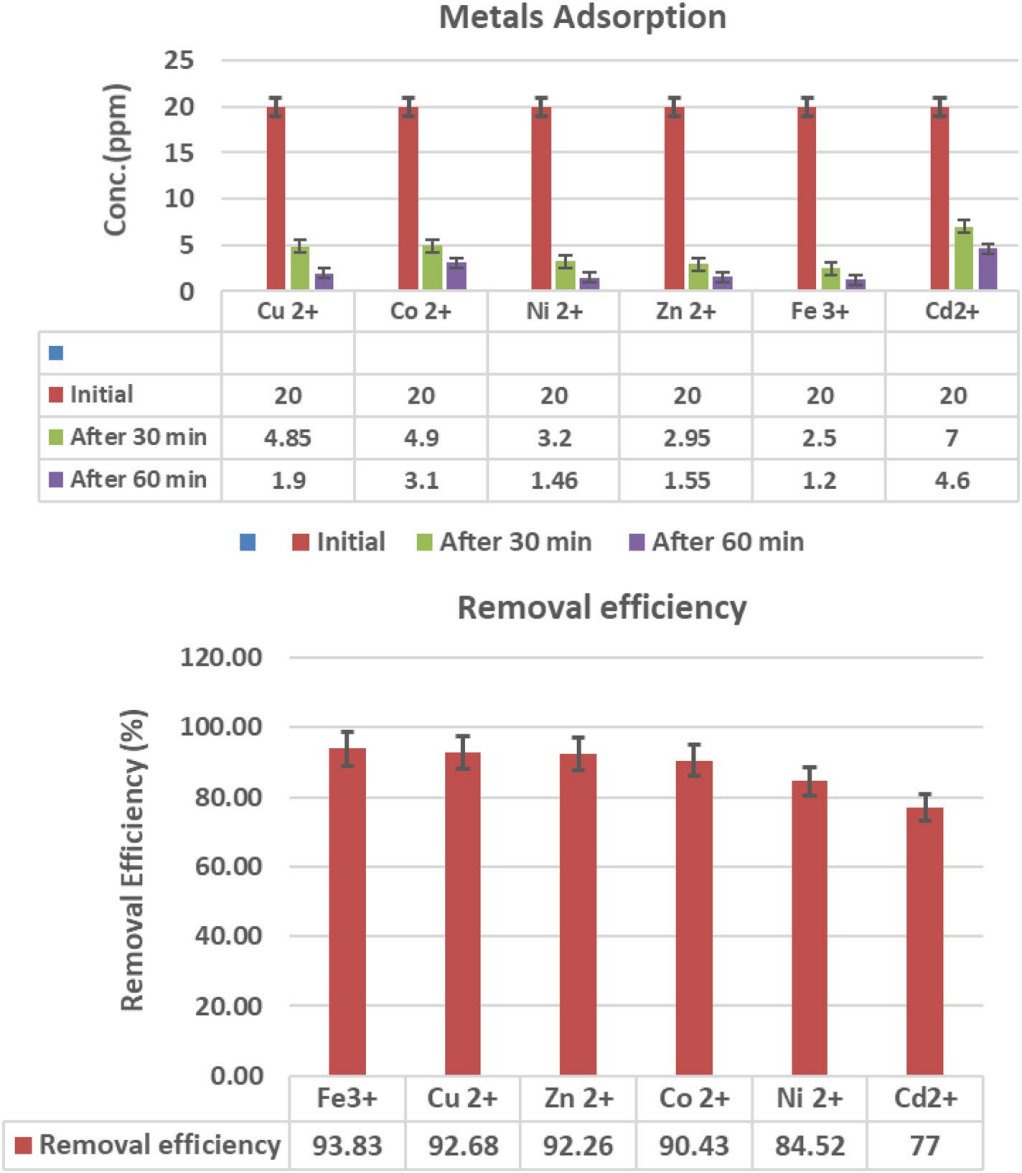
The top chart shows flame atomic adsorption of heavy metals after 30 and 60 min of treatment with 0.05 gm of PAK, which describes the remaining concentration of each metal ion, and the down chart shows removal efficiency % of PAK.

Adsorption removes heavy metals from a solid surface, creating equilibrium by keeping adsorbed heavy metal concentrations in water constant. The conventional interaction is chemical adsorption to attain higher adsorption capacity for heavy metal removal. The projected chemical adsorption pathway is depicted in Fig. 9. The maximum adsorption capacities (Cd^2+^ < Ni^2+^ < Co^2+^< Zn^2+^< Cu^2+^ <Fe^3+^) were within the equilibrium time approaching 1 hour, with AE 93.83%, 92.68%, 92.26%, 90.43%, 84.52%, and 77% for Fe, Cu, Zn, Co, Ni, and Cd, respectively.

**Figure 10:**
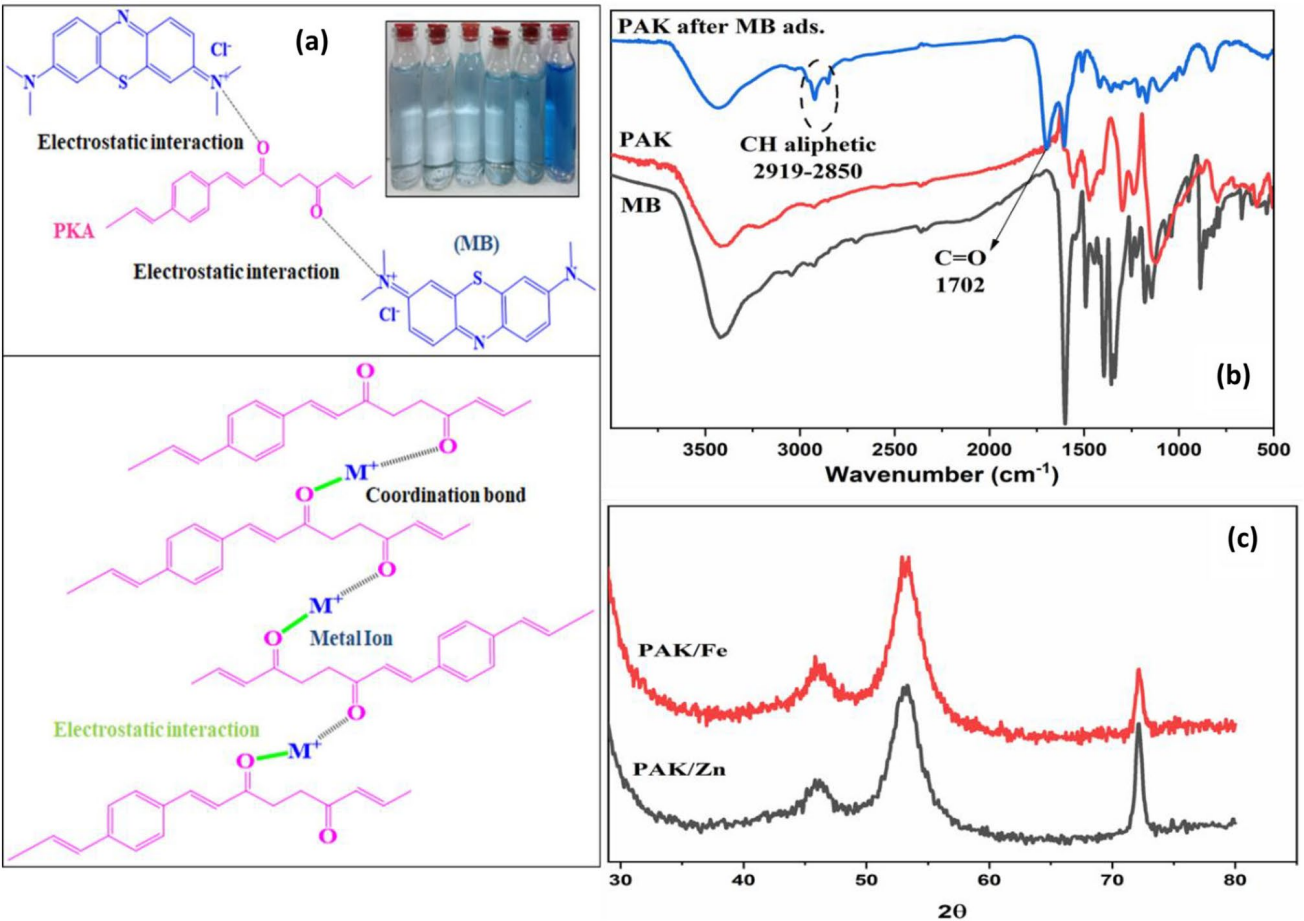
(**a**) Schematic illustration of MB adsorbed and the reaction mechanism of heavy metals removal from wastewater using PAK polymer. (**b**) FT-IR of PAK after MB Adsorption, (**c**) X-ray of PAK after Zn and Fe removal.

The polymer's structure shape, surface area, and strong disposal capacity make it a better adsorbent for heavy metal removal. Because we have ketone groups in the polymer main chain, removing metal ions by chemical adsorption involved chelation ion exchange, which resulted in electrostatic contact between metal ions and polymer. Figure 10 displays the results of the PAK's XRD test after Zn^2+^ and Fe^3+^ metals adsorption; new diffraction peaks at 2θ = 46°, 53°, and 72°, corresponding to Zn and iron metals, occurred when comparing the XRD patterns of PAK before and after adsorption. This proves that chemisorption is the process used by PAK to bind Zn^2+^ and Fe^3+^^72,73^.

After MB adsorption, the FT-IR spectrum of PAK in Fig. 10 shows distinct peaks at 2919 and 2850 cm^-1^, which are indicative of C-H stretching and, more precisely, the vibrations of the aliphatic -CH_3_ group in MB dye and CH_2_ in PAK. In addition, the peak at 1702 cm^-1^ indicates the C=O functional group, while the peak at 1602 cm^-1^ is indicative of conjugation. Since the non-covalent interactions of carbonyl oxygens and the faces of aromatic surfaces are repulsive as more rings are added to the aromatic surface, the enhancement of the carbonyl group suggests that the mechanism of adsorption occurred via stacking and electrostatic attraction. As more rings are added to the aromatic surface, the carbonyl group's dipole moment increases, causing it to appear in the spectrum^74,75^.

### Selectivity of the PAK

**Figure 11:**
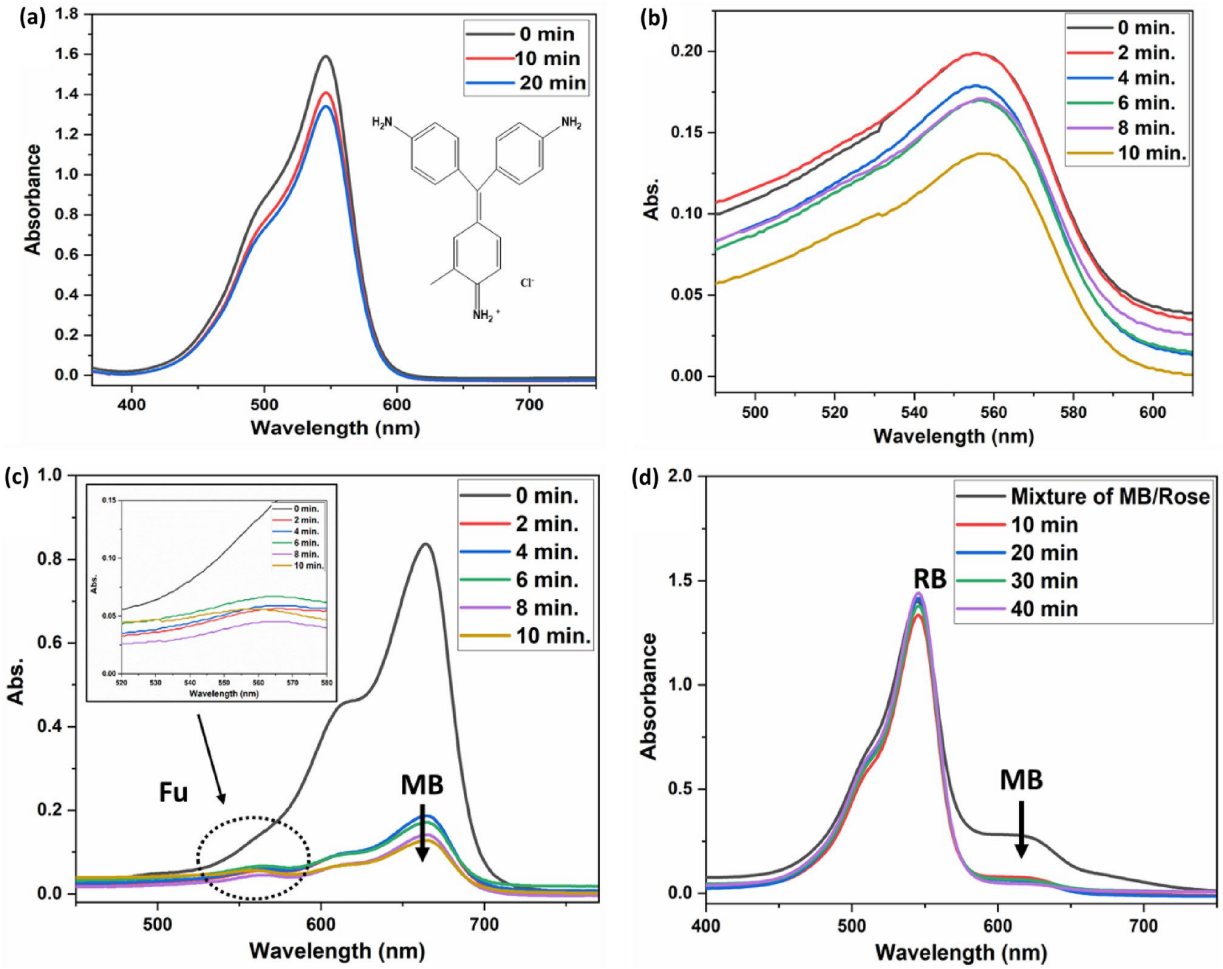
(**a**) Show UV–Vis spectra of 40 ppm Fu 0.05 mg PAK with pH 10**.** (**b**) UV–Vis spectra of 20 ppm Fu 0.05 mg PAK with pH 7.2. (**c**) UV–Vis spectra of mix 20 ppm MB/20 ppm Fu 0.05 mg PAK with pH 8. (**d**) UV–Vis spectra of mix 20 ppm MB/20 ppm RB 0.05 mg PAK with pH 8.

Basic fuchsine (Fu) is another type of cationic dye. The wavelength of basic fuchsine (Fu) dye is 544 nm, and the cloud electron mobility inside the molecule causes the positive charge to be dispersed symmetrically throughout the molecule. Adsorption of the fuchsine dye Fu is carried out to ascertain the quantity of Fu dye that is adsorbed and evaluate its removal compared to MB. We utilize 0.05 gm of PAK in conjunction with 200 ml of dye solution with concentrations of 40 ppm, pH 10, and 20 ppm, pH 7.2. In accordance with the findings of the adsorption trials presented in (Fig. 11a,b- Table S1), the adsorption effect of the Fu dye was significantly lower than that of the MB dye, reaching only about 31% when compared to the MB dye. The calibration curves of MB dye, Fu dye, and the mixture of the two are created to determine and monitor the concentration of the dyes solution (Fig. S2). Additionally, the adsorption behavior of PAK towards the mixture of MB and Fu is examined. Through the incorporation of 0.05 mg of adsorbent into a binary mixture consisting of 100 ml of MB solution at 20 ppm and 100 ml of Fu solution at 20 ppm. According to the findings (Tables S1 and S2 and Fig. 11c), the two different types of molecular dyes, (MB) and (Fu), are hydrophilic and cationic, respectively. Furthermore, the solution of a single (MB) dye fades more quickly than the solution of a single (Fu) dye, which demonstrates that PAK can distinctly adsorb (MB) molecules. This consequence is very apparent in the combination. When the percentage of MB in the solution decreases from 50 to 8%, the number of adsorbed dyes decreases significantly and almost reaches its maximum level. This results in the production of a very similar colour to the Fu dye with a small concentration. Because of the nature of MB dye, which PAK rapidly adsorps, the resultant adsorbent demonstrated selective adsorption to MB rather than Fu. The explanation for this selectivity could be due to the structure of Fu dye and the positive charge that is dispersed throughout the structure, as an organic dye adsorption process consists of two mechanisms, electrostatic strength and diffusive contacts, and the molecule's low absorptivity is owing to its size and spatial distribution. Large molecules cannot efficiently pass through the small pores, and the subsequent aggregation and buildup of these massive molecules at the top surface of the small pores lead to pore clogging, a restriction of additional mass transfer, and insufficient adsorption^76^.

Additionally, we examine the selectivity of the MB dye in the presence of an anionic dye called rose bengal RB, which has a 550 nm UV absorbance Fig. 10d. Due to the abundance of carbonyl groups in the adsorbent, the cationic dye MB could be adsorbed on the negatively charged adsorbent due to electrostatic attraction; however, the anionic dye RB found it challenging due to electrostatic repulsion. To investigate the adsorbent's preferential adsorption to the MB dye, 200 mL of MB/RB mixed solution (pH = 10; MB and RB concentrations were both 20 ppm) was used for selective research. Figure 11d illustrates how the adsorbent reduces the quantity of MB dye while maintaining a practically constant concentration of the other dye. The adsorbent quickly absorbs the MB dye, indicating that effective selectivity is brought about by the PAK adsorbent's negative zeta potential.

### Reusability of PAK adsorbent

**Figure 12:**
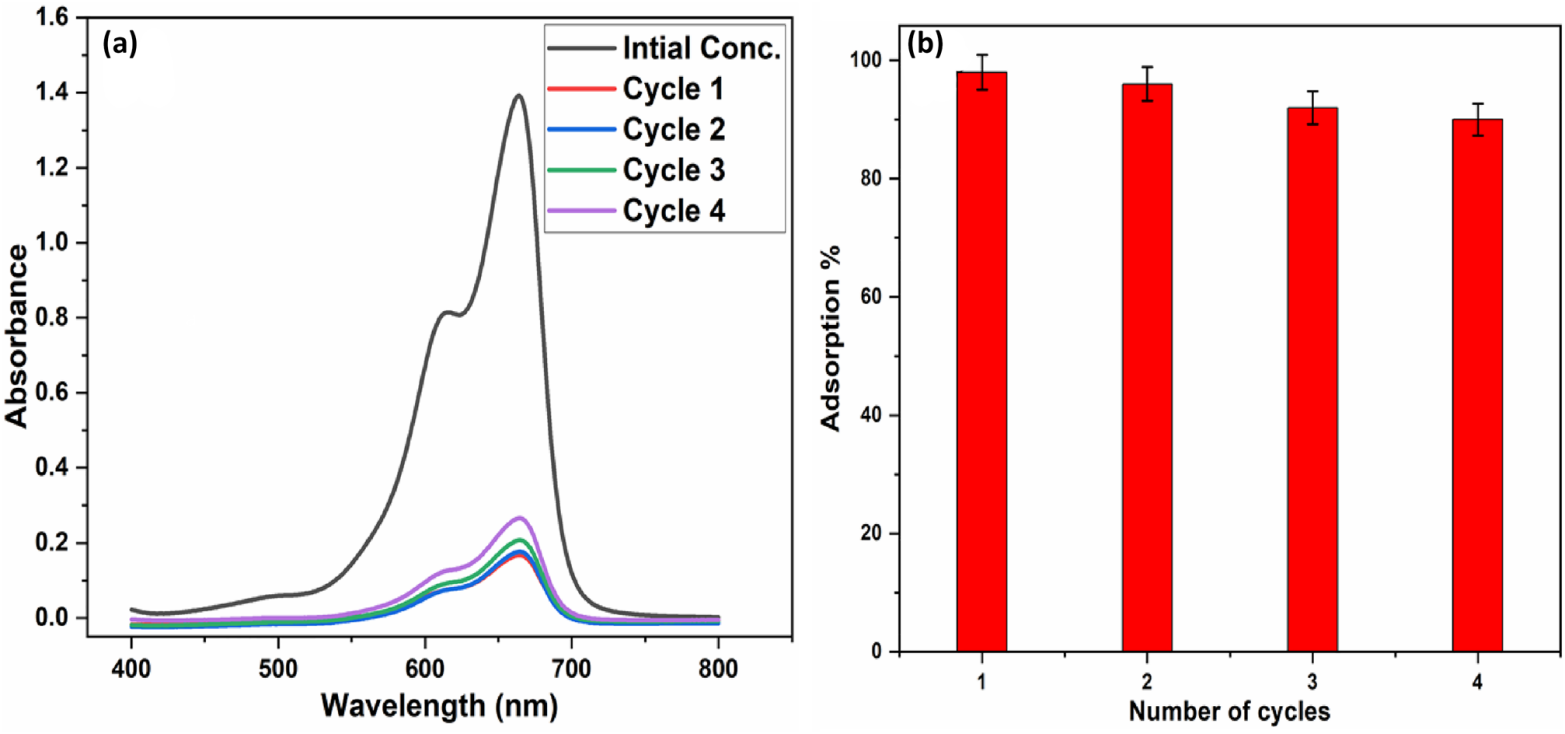
(**a**) Reuse of PAK adsorbent after 4 cycles, and (**b**) Diagram of the Reusability of PAK.

One of the most significant potential uses for wastewater treatment adsorbents is regeneration and reuse. Figure 12 shows the dye adsorption capabilities over the four cycles of desorption testing using ethanol to regenerate the adsorbent. As the number of regeneration cycles increased, the MB's adsorption capabilities rapidly declined. The ineffective regeneration sites on the adsorbent account for the low adsorption capacity following regeneration^77^. Figure 12 demonstrates that the adsorption efficiency for MB declined from 98 to 90% after four reuse cycles. In addition, neither adsorption nor desorption significantly altered the bulk of the adsorbent. As a result, PAK has a strong adsorption capacity and is recyclable for dye removal.

## Conclusion

PAK was synthesized using a simple and convenient condensation polymerization technique with alternating ketone groups at one and four-distance locations. The polymer was characterized by FTIR, SEM, XRD, TGA, fluorescence, and UV-Vis spectroscopy. The MB adsorption on PAK was considerably impacted by process factors (pH, amount of PAK, and MB starting concentration). At pH 10 and room temperature, the highest removal proficiency of 96% was attained. Furthermore, the MB adsorption on the produced polymer fits perfectly with the pseudo-2nd-order kinetic and Freundlich isotherm models. Moreover, it is a unique and environmentally friendly adsorbent that can be made in large quantities at a low cost using a simple and convenient one-pot condensation process. Furthermore, the PAK's chelation-mediated adsorption selectivity for heavy metal ions, especially Fe^3+^, makes it a viable addition to the existing adsorbents for removing Fe^3+^ metal and MB dye from wastewater.

## Supplementary Information

Supplementary Information.

## Data Availability

All data generated or analyzed during this study are included in this published article [and its supporting information files].
