# Supplementary material for: Synthesis, characterization and application of high adsorption performance of novel 1,4-polyketone
Source: Sci Rep. 2022 Sep 29;12:16317. doi: 10.1038/s41598-022-20686-7 (PMC9523028; doi:10.1038/s41598-022-20686-7)
Supplement: Supplementary file 1 — Supplementary Information. [file 41598_2022_20686_MOESM1_ESM.docx]

**Synthesis, characterization and application of High adsorption performance of Novel 1,4-polyketone**

Marwa M. Sayed^1,*^, Islam S Abd El-Hamid^2,3^, Haitham M. El-Bery^4^, Mostafa Farrag^5^, Alaa K. Abdelhakiem^6^**,** Kamal I. Aly^2,*^

^1^Chemistry Department, Faculty of Science, the New Valley University, El-Kharja 72511, Egypt.

^2^Polymer Research laboratory, Chemistry Department, Faculty of Science, Assiut University, Assiut, 71516, Egypt.

^3^Assiut Cement Company (Cemex Egypt) 18^th^, Assiut- Elwadi Road, Assiut-Egypt.

^4^Advanced Multifunctional Materials Laboratory, Chemistry Department, Faculty of Science, Assiut University, Assiut, 71515, Egypt

^5^Nanoclusters and Photocatalysis Laboratory, Chemistry Department, Faculty of Science, Assiut University, 71515 Assiut, Egypt.

^6^Department of Pharmacy Practice and Clinical Sciences, University of Texas at El Paso School of Pharmacy, El Paso, Texas, USA.

**Corresponding Authors:**

Professor Kamal I Aly

Email: [Kamalaly@aun.edu.eg](mailto:Kamalaly@aun.edu.eg)

Marwa Sayed

Email: marwa.m@sci.nvu.edu.eg

**
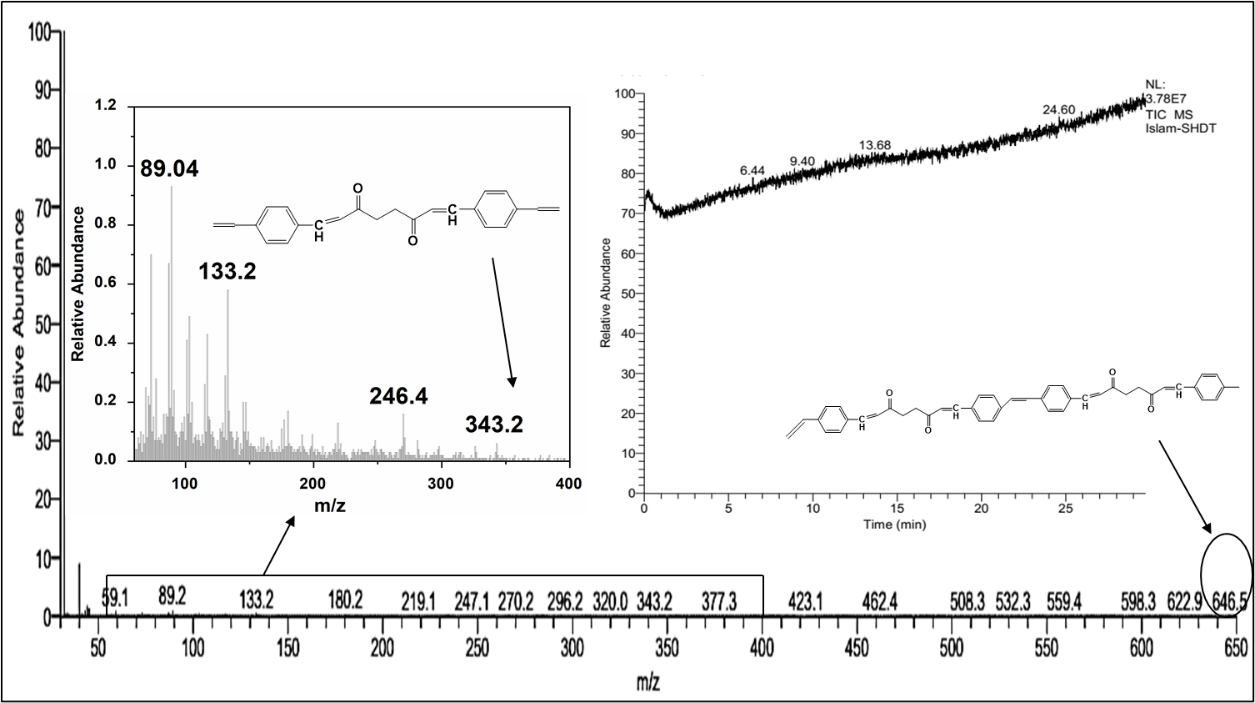
**

**Figure S1:** Top view entirety and time windows between 0-29.7 min of the Pyrolysis-GC/Mass chromatogram of PAK pyrolyzed at 700 °C.


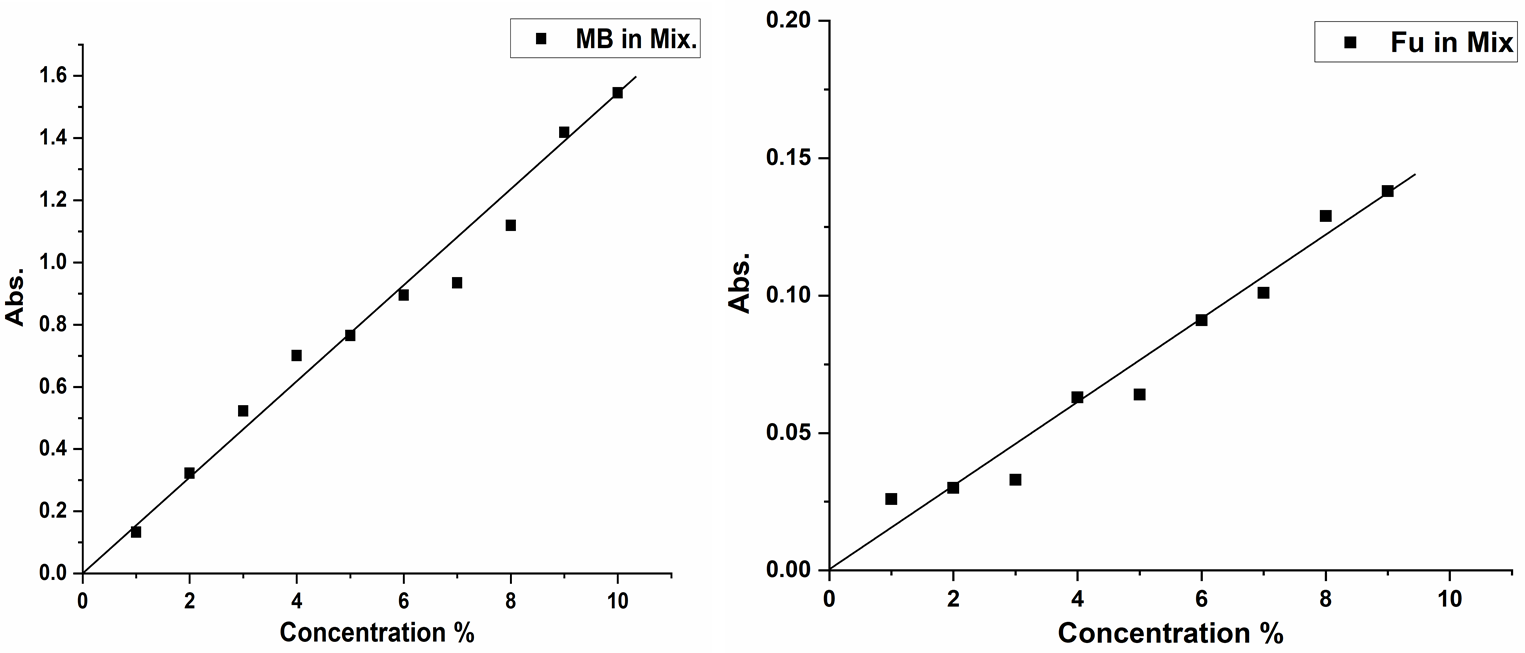


**Figure S2.** Calibration curves of MB and Fu dyes in mixture.

**Table S1.** Adsorption efficiency (AE%) of (Fu) dye solution 20 ppm with PAK adsorbent.

| **Time (min.)** | **AE% of Fu** |
| --- | --- |
| **2** | **0 %** |
| **4** | **10 %** |
| **6** | **14 %** |
| **8** | **14 %** |
| **10** | **31%** |

**Table S2.** Percent (%) of (MB) and (Fu) dye solution (20 ppm each) in a binary mixture with PAK adsorbent.

| **Time (min.)** | **% of MB** | **% of Fu** |
| --- | --- | --- |
| **2** | **12 %** | **50 %** |
| **4** | **12 %** | **36 %** |
| **6** | **11 %** | **30 %** |
| **8** | **9 %** | **29 %** |
| **10** | **8 %** | **22 %** |
